# Coastal canaries

**DOI:** 10.1017/cft.2026.10038

**Published:** 2026-06-18

**Authors:** Gerd Masselink, Eli D. Lazarus, Ian Townend

**Affiliations:** 1 Centre for Coastal and Ocean Processes and Engineering, University of Plymouth, UK; 2 School of Geography and Environmental Science, University of Southampton, UK; 3 School of Ocean and Earth Sciences, University of Southampton, UK

**Keywords:** coastal erosion, coastal zone management, sea-level rise, shoreline management plans

## Abstract

The winter storms of January and February 2026 caused severe coastal damage along southwest England, reviving memories of the destructive 2013/14 winter and exposing persistent weaknesses in UK coastal resilience policy. Using the Slapton Sands gravel barrier system, in Devon, as a case study, this article argues that repeated damage to the village of Torcross and the A379 road section that runs along the barrier illustrates a broader national cycle of escalating coastal risk despite decades of scientific understanding and strategic planning through Shoreline Management Plans (SMPs). Although none of the winter 2026 storms were individually extreme, their rapid succession, sequencing, and coincidence with spring tides caused substantial erosion, infrastructure collapse, and property damage, highlighting the cumulative threat of compound events. The case study exemplifies how defended coastlines and transport corridors can function as “single points of failure,” where disruption produces cascading social and economic consequences. While SMPs acknowledge the eventual need for “Managed Realignment,” implementation remains slow, politically contentious, and operationally vague, with policy often defaulting to short-term “Hold the Line” responses. This creates an adaptation deficit between national ambitions for resilience and practical action on the ground. We emphasise that transformational adaptation – including managed realignment, infrastructure relocation and community transition – requires coordinated national leadership, statutory support, a consistent approach and socially just frameworks that prioritise participation and fairness. Current local and national governance structures remain insufficient to meet the pace and scale of climate-driven coastal change. Torcross, like many vulnerable coastal communities, serves as an early warning of broader systemic failure: sea-level rise and intensifying coastal hazards will increasingly force the UK to confront difficult choices about protection, retreat and relocation.

## Graphical abstract

**Figure:**
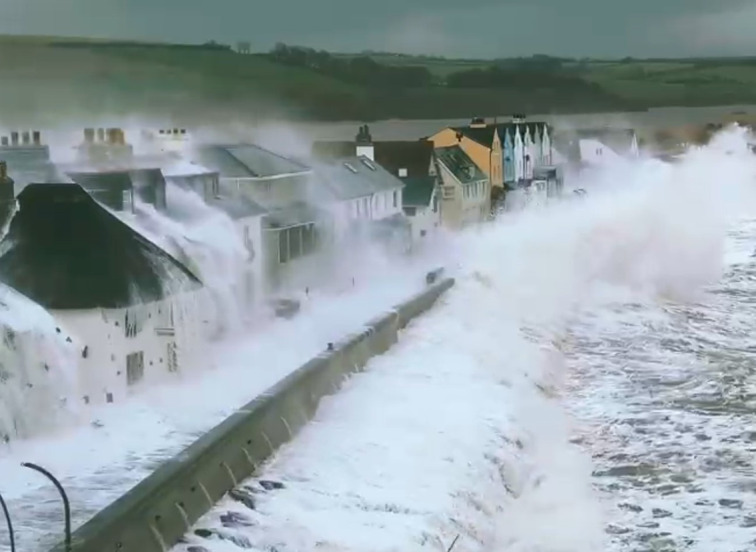

## Impact statement

The winter storms of early 2026 highlighted the continuing vulnerability of UK coastlines despite decades of coastal planning. The Slapton Sands barrier and the village of Torcross in Devon demonstrate how repeated storm events can damage homes, infrastructure, and the A379 transport corridor, exposing a widening gap between national resilience ambitions and practical adaptation on the ground. Although none of the storms were individually exceptional, their rapid succession and alignment with spring tides caused severe erosion and disruption, underlining the growing threat from compound coastal events. The case study also shows how defended coastlines can become systemic “single points of failure”, generating wider social and economic impacts when defences fail. While Shoreline Management Plans increasingly recognise the need for managed realignment, delivery remains slow, politically sensitive, and poorly coordinated. Smaller rural communities are often disproportionately affected and require credible future scenarios and adaptation pathways to support local engagement and decision-making. Identifying settlements that should begin adaptation planning now could help communities use evidence-based narratives to secure government support. Effective adaptation will require nationally coordinated approaches to managed realignment and relocation that are economically viable, socially acceptable and centred on community participation, fairness and people’s attachment to place. Torcross represents an early warning for coastal communities across the UK facing rising seas, intensifying hazards and difficult decisions over protection, retreat and relocation.

In January 2026, three named Atlantic storms – Goretti, Ingrid, and Chandra – significantly impacted the Atlantic coast of Europe with damages along the coast of SW England reminiscent of the 2013/14 winter storm season (Masselink et al., 2016; Castelle et al., 2017). While the UK government has set out a forward-looking ambition to “create a nation more resilient to future flood and coastal erosion risk” (Defra, 2020) – an ambition echoed in the National Flood and Coastal Erosion Risk Management Strategy for England (Environment Agency, 2020) – the physical and economic consequences of the January storms feel like *déjà vu* . There is no shortage of science, engineering, planning and local expertise underpinning the 24 Shoreline Management Plans (SMPs) that wrap around England and Wales. So why does the UK appear caught in a cycle of escalating risk associated with coastal hazards?

**Figure 1.**
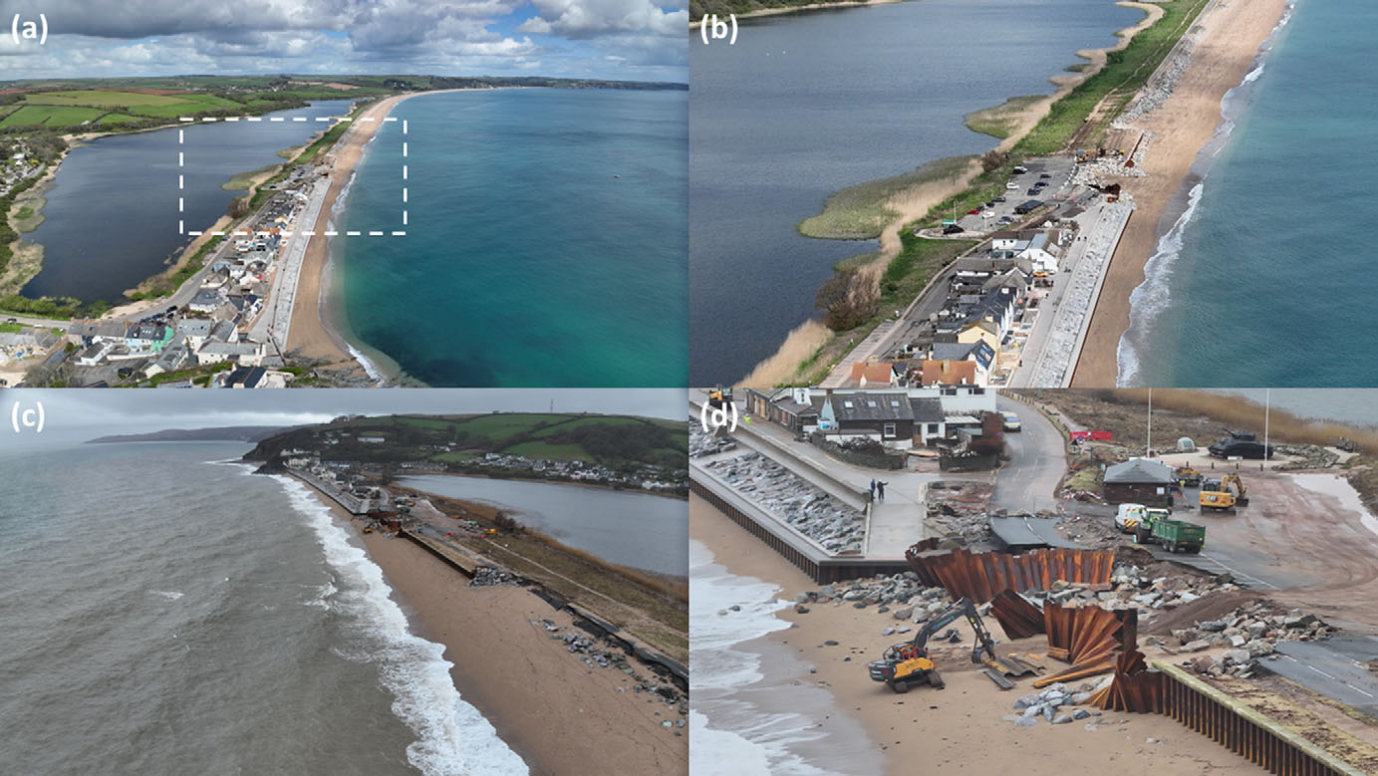
(a) View towards the north of Torcross and the Slapton Sands barrier; (b) close-up of boxed section in (a) showing the northern end of the seawall and the damage to the A379; (c) view towards the south of the damaged road section and Torcross; and (d) close-up of the collapsed road section and northern end of the seawall (photos: Dr Tim Scott). Figure 1. long description.

Consider the Devon village of Torcross (Figure 1). Situated on the gravel barrier of Slapton Sands, the village is connected to its coastal neighbours to the north – Slapton, Strete, and Dartmouth – by the A379, a road known as “the Slapton Line” for the way it traces the barrier crest. Torcross is fronted by a seawall, built after winter storms in 1979 almost destroyed the village. When a northern section of the A379 was eroded in 2001, the resulting disruption and road realignment prompted discussions through the Slapton Line Partnership about the sustainability of the road with climate change and sea-level rise. A dozen years later, the winter storms of 2013/14 caused extensive damage to Torcross and the A379, along with substantial beach lowering. Two years after that, following the 2015/16 winter, beach levels at Torcross were so depleted that the seawall began leaning seawards, necessitating £3M in emergency repairs. In 2018, a March weather system nicknamed the “Beast from the East” combined with the named storm Emma to further damage the A379 enough to require a second realignment.

Then after a period of relative meteorological quiet came the trio of storms of January 2026. According to data from the Start Bay directional wave buoy, none was exceptional: Goretti was a 1:0.25 year event; Ingrid was a 1:5 year event; and Chandra was a 1:1 year event (CCO, 2026). More unusual was the arrival of these storms in such rapid succession, and the fact that two of the storms coincided with spring high tide conditions. Their combined impact was profound. After the peak of Ingrid, on 23 January, sheet piling in front of the seawall – part of the emergency repair in 2015/16 – was exposed to a depth of 2 m. Beach gravel was propelled over the seawall directly against houses, damaging windows, doors, walls, and roofs. So much of the protective beach along the southern reach of the A379 was lost that in the first week of February, when an otherwise unremarkable set of energetic wave conditions (*H_s_* < 3 m) arrived from the east, a 200-m section of the A379 was completely washed out.

The A379 at Torcross, the coastal rail line at Dawlish (Oates, 2026), and countless other infamous sections of coastal road and rail track in the UK and around the world that fail with extreme weather, exemplify “single points of failure” in critical infrastructure networks (Aldabet et al., 2022). Recent UK Climate Change Risk Assessments (Dawson et al., 2016; Betts et al., 2021) list systemic risks to transportation networks from flooding – with cascading failures of dependent services – as an area of particular urgency for national climate adaptation. The assessments cite “an underlying need to assess the impact of single points of failure more broadly” because “a paucity of data is restricting progress in these areas” (Betts et al., 2021). Even the Joint Committee on the National Security Strategy has called out an “adaptation deficit” (JCNSS, 2022) in the extent to which the UK has addressed systemic risk in climate-change impacts on national critical infrastructure.

In England and Wales, SMPs are the main policy lever for coastal adaptation, but they are non-statutory instruments (Stojanovic, 2025). For 20-, 50- and 100-year horizons, or “epochs,” Shoreline Management Plans evaluate four policy options: No Active Intervention, Managed Realignment, Hold the Line, and Advance the Line. For the policy unit that includes Torcross, the current advice is Hold the Line to 2055 (i.e., maintaining or replacing the current seawall) and Managed Realignment from 2055 to 2105. The unit north of Torcross, where the A379 runs atop the barrier, recommends Managed Realignment from 2025 to 2055, and from 2055 to 2105. A recent national-scale analysis suggests that in England, approximately a third of the shoreline currently designated as Hold the Line is likely to see increasing pressure to realign (Sayers et al., 2022). The latest National Coastal Erosion Risk Mapping exercise for England (NCERM2, 2025) highlights southwest England as one of the regions – together with Yorkshire, the Humber, and east England – most at risk from coastal erosion. The mapping exercise assumes that recommended Hold the Line actions will be funded and implemented through the repair and maintenance of existing coastal protections, limiting the properties at risk to 5,200 and 19,700 by 2055 and 2105, respectively. But without future investment to manage coastal erosion and accommodate sea-level rise, the total number of properties in areas at risk of coastal erosion is projected to increase these numbers almost ninefold by 2055, and more than fivefold by the end of the century (NCERM2, 2025). Bad as that sounds, these potential impacts from coastal erosion are far outstripped by those associated with coastal flooding (Sayers et al., 2022).

Immediately after the winter closure of the A379, a lively debate started in the Start Bay region about whether the A379 should be repaired or abandoned, with an alternative route developed to keep Torcross connected to its neighbours along the Slapton Line. Over 39,000 people signed a petition urging the government to support communities affected by erosion along the A379. Devon County Council, who are responsible for the road, announced that preliminary estimates to rebuild the Slapton Line are currently costed at around £18M – not including the cost of any sea defences. To put that figure in context: the Environment Agency announced on 28 January 2026 that coastal communities in England are set to benefit from £30M to address eroding shorelines by means of innovative adaptive measures, not including conventional coastal protection (EA, 2026). Under the EA’s new Coastal Adaptation Pilots (Foley, 2026), £18M has already been allocated to projects across the East Riding of Yorkshire, Norfolk, and Suffolk to develop innovative approaches to coastal transition, including selective property purchases.

However, more recently, attention has shifted to whether the seawall protecting Torcross requires strengthening, as energetic waves impacting the seawall during high tide are causing the houses at the sea front to physically tremble, and residents are justifiably worried about their personal safety. The BBC reported in May 2026, three months after the road collapse, that the Environment Agency had approved a £19.8M funding scheme to install rock armour in front of the Torcross seawall (Woodling and Thorpe, 2026). So, while the future of the A379 remains in question, “saving the village” has been considered a priority, even though its “rescue” will inevitably lead to the loss of the beach in front of the seawall – part of a global trend on developed beaches (Pilkey and Cooper, 2014).

In comparison with the cost of rebuilding the A379 or installing rock armour in front of the Torcross seawall, the direct cost of repairs to a section of rail line at Dawlish – which the 2013/14 winter storms turned into a “Peruvian rope bridge” (BBC, 2014), compounded by a landslip to the south, at Teignmouth – are larger by an order of magnitude. The BBC recently reported that since 2014, four phases of a five-part refurbishment have cost taxpayers £165M; a fifth phase was “paused” by the Labour government in the summer of 2025 based on expense (Oates, 2026). A report by the Devon Maritime Forum estimated indirect costs of the 2014 closure to the economies of Devon and Cornwall ranged between £60M and £1.2B (BBC, 2015). While the next phase of works is uncertain, the Dawlish segment remains a latent “single point of failure.”

The potential realignment of many defended sections of coast, typically where population density is low, reflects the long-term national ambition for a more resilient coastline. Yet there remains an operational gap between ambition and action (Townend et al., 2021; Van der Plank, 2024). Implementation of Managed Realignment, for example, is currently five times too slow to meet the stated 2030 targets (Stojanovic, 2025). A policy shift from Hold the Line to Managed Realignment or even No Active Intervention represents a transformational change that would likely have to include the relocation of some coastal communities, but there is little guidance regarding how such a transformation would be supported (Sayers et al., 2022). Moreover, policy transition from protection to realignment is often resisted by affected communities (Buser, 2020) and can carry high political costs for elected officials (Brown et al., 2023).

The epoch-based framework of SMPs has had less than the desired effect in spurring political responsiveness and innovative coastal adaptation: decisions tend to get postponed to the future, and there is a lack of clarity regarding which specific actions will be taken to transition to a new policy, and when (Stojanovic, 2025). At the most local level, only 15% of coastal planning authorities have designated a Coastal Change Management Area in their Local Plans (Kirby et al., 2021). Even so, local stakeholders are likely to find that their capacity to initiate transformational change is structurally limited (Van der Plank, 2024). A 2011 report by the Department for Environment, Food and Rural Affairs on climate-resilient infrastructure (DEFRA, 2011) laid out the sectoral cooperation needed to address infrastructure vulnerability: national government; local authorities; infrastructure owners, operators and regulators; private-sector engineering firms specialising in adaptation; and re/insurers. Successful engagement across so many facets and so many institutional and physical scales would be a monumental achievement. But the list of players also signals the precarity of such an endeavour.

This is not a new revelation. The need for some communities and infrastructure to relocate away from the shore was recognised from the outset of the original SMP process three decades ago. Relatively small, rural communities tend to be the ones most affected, and could benefit from storylines and road maps of future scenarios credible to them (Robinson et al., 2022; Townend et al., 2025; Melbourne-Thomas et al., 2026) to help them engage with adaptation trajectories (Leafe et al., 1998). Legal, scientific, engineering, and political constraints on possible ways forward should be made clear, but should not discourage proactive solutions. The drivers of any adaptive intervention should be community-owned bodies working alongside a regional or national adaptation policy unit. A starting point might be to identify all the towns and villages that should push now towards an adaptation plan; much of this work has already been done in the non-statutory SMPs. The coastal research community could identify areas that need an adaptation plan and estimate the degree of urgency. Promoting the results as narratives, through a range of media channels, may enable local communities to then use the evidence to press the government for support, especially where urgency is deemed high.

Community participation in the design and implementation of viable adaptation pathways must be supported by a national-scale approach to managed realignment and relocation that is robust, economically viable and socially acceptable. Any planned relocation options must be seen as a fundamentally social process, recognising people’s attachment to their homes, and prioritising participation and fairness (Möller, 2026). Some of these aspects have been recognised in a recent House of Commons committee report (EFRA, 2026) focusing on the impact of coastal erosion on communities. Apart from acknowledging that coastal erosion is already causing profound and distressing disruption to people’s lives, the report further highlights the lack of resources for adaptation and consistent national guidance.

Sea-level rise will force the UK into some kind of transformational coastal adaptation, just as it will along human-altered coasts around the world (McNamara et al., 2023). For now, a resilient coastal future for the UK seems a distant aspiration. Beachside communities like Torcross are perched like coastal canaries, indicators of functional conditions at the hazard interface. After yet another winter battering, these canaries are telling us all we need to know.

## Data Availability

Not applicable.

## Long descriptions

### Figure 1. Long description

A four-panel series of aerial photos by Dr Tim Scott.

* Panel a: A wide aerial view looking North. To the West is a freshwater ley, and to the East is the sea. A thin shingle barrier beach separates them, carrying the A3 79 road and a line of houses at Torcross. A white dashed box highlights the northern end of the village.

* Panel b: A close-up of the boxed area from panel a. The paved seawall ends abruptly, and the adjacent A3 79 road is severely fractured with large sections of asphalt missing. Construction debris and exposed earth are visible where the road has collapsed into the shingle.

* Panel c: An aerial view looking South toward Torcross. The perspective shows the long stretch of the Slapton Sands barrier. The foreground reveals a significant breach where the road has been undercut by wave action, leaving a jagged edge of broken pavement.

* Panel d: A high-detail close-up of the damage site. Rusty corrugated metal sheet piling is bent and twisted outward toward the beach. An orange excavator is positioned on the sand, working near the base of the collapsed road. Large granite boulders are scattered around the breach to provide temporary coastal defense.

Navigate back to Figure 1..
