# Supplementary material for: Coastal canaries
Source: Camb Prism Coast Futur. 2026 Jun 18;4:e19. doi: 10.1017/cft.2026.10038 (PMC13458089; doi:10.1017/cft.2026.10038)
Supplement: Peer review history

# Reviewer report: Review: Coastal canaries — R0/PR2

**DOI:** 10.1017/cft.2026.10038.pr2

**Authors:** Iris Moeller

**Affiliations:** Geography, Trinity College Dublin, Ireland

I congratulate the authors on putting together a thoroughly researched, evidenced, topical, and highly societally relevant perspectives paper. The paper deals with the challenge of coastal management in the UK context, and particularly coastal erosion and flood protection, at a time when pressures on coastal systems are more intense than ever and recognition of the way they function is much needed. It is well evidenced, highly factual, but written in a narrative style that makes the facts tell a particularly compelling story in a way that I hope a will allow a wide readership. It is also extremely readable, written succinctly, to the point, and in a well-structured and rounded way. It is important to publish this paper for a number of reasons but particularly given the pivotal moment that we are facing as the coastal management mistakes of the past decades, escalating costs, and accelerating sea level rise alongside reconfigured weather patterns make themselves felt acutely.

I have one small – but I think important – suggestion to the authors to ensure that the paper reflects others’ efforts to draw attention to similar issues: I was surprised to see the below absent from the references and suggest that the authors add the well-researched and articulated publication by Pilkey and Cooper, published in 2014, that has, in my opinion, been given too little attention but makes very similar points to this paper:

Pilkey, O.H., and Cooper, J.A.G. 2014. The Last Beach. Duke University Press, 237pp.

I spotted one typo: Line 44 on page 4 should be ‘adaptation’ and not ‘adaption’.

# Editor report: Recommendation: Coastal canaries — R0/PR3

**DOI:** 10.1017/cft.2026.10038.pr3

**Affiliations:** Geography, Cambridge University, United Kingdom of Great Britain and Northern Ireland

Minor revision

# Editor report: Decision: Coastal canaries — R0/PR4

**DOI:** 10.1017/cft.2026.10038.pr4

**Affiliations:** Geography, Cambridge University, United Kingdom of Great Britain and Northern Ireland

No accompanying comment.

# Editor report: Recommendation: Coastal canaries — R1/PR6

**DOI:** 10.1017/cft.2026.10038.pr6

**Affiliations:** Geography, Cambridge University, United Kingdom of Great Britain and Northern Ireland

See HE comments. Accept.

# Editor report: Decision: Coastal canaries — R1/PR7

**DOI:** 10.1017/cft.2026.10038.pr7

**Affiliations:** Geography, Cambridge University, United Kingdom of Great Britain and Northern Ireland

No accompanying comment.
